# Why Senescent Cells Are Resistant to Apoptosis: An Insight for Senolytic Development

**DOI:** 10.3389/fcell.2022.822816

**Published:** 2022-02-16

**Authors:** Li Hu, Huiqin Li, Meiting Zi, Wen Li, Jing Liu, Yang Yang, Daohong Zhou, Qing-Peng Kong, Yunxia Zhang, Yonghan He

**Affiliations:** ^1^ Department of Geriatrics, The Second Affiliated Hospital of Hainan Medical University, Haikou, China; ^2^ State Key Laboratory of Genetic Resources and Evolution, Kunming Institute of Zoology, Chinese Academy of Sciences, Kunming, China; ^3^ College of Basic Medicine and Life Sciences, Hainan Medical University, Haikou, China; ^4^ Department of Endocrinology, The Third People’s Hospital of Yunnan Province, Kunming, China; ^5^ Lab of Molecular Genetics of Aging and Tumor, Medical School, Kunming University of Science and Technology, Kunming, China; ^6^ Department of Pharmacodynamics, College of Pharmacy, University of Florida, Gainesville, FL, United States

**Keywords:** aging, apoptosis, resistance, senescent cell, senolytic

## Abstract

Cellular senescence is a process that leads to a state of irreversible cell growth arrest induced by a variety of intrinsic and extrinsic stresses. Senescent cells (SnCs) accumulate with age and have been implicated in various age-related diseases in part via expressing the senescence-associated secretory phenotype. Elimination of SnCs has the potential to delay aging, treat age-related diseases and extend healthspan. However, once cells becoming senescent, they are more resistant to apoptotic stimuli. Senolytics can selectively eliminate SnCs by targeting the SnC anti-apoptotic pathways (SCAPs). They have been developed as a novel pharmacological strategy to treat various age-related diseases. However, the heterogeneity of the SnCs indicates that SnCs depend on different proteins or pathways for their survival. Thus, a better understanding of the underlying mechanisms for apoptotic resistance of SnCs will provide new molecular targets for the development of cell-specific or broad-spectrum therapeutics to clear SnCs. In this review, we discussed the latest research progresses and challenge in senolytic development, described the significance of regulation of senescence and apoptosis in aging, and systematically summarized the SCAPs involved in the apoptotic resistance in SnCs.

## Introduction

Aging is a time-dependent functional decline that affects most living organisms. It is characterized by a progressive loss of physiological integrity, causing impaired function and increased vulnerability to death (López-Otín et al., 2013). Aging results from the time-dependent accumulation of a wide range of molecular and cellular damage over time. In humans, aging increases the risk of various age-related diseases. In 2013, López-Otín et al. identified and categorized the cellular and molecular hallmarks of aging, including genomic instability, telomere attrition, epigenetic alterations, loss of proteostasis, deregulated nutrient sensing, mitochondrial dysfunction, cellular senescence, stem cell exhaustion and altered intercellular communication (López-Otín et al., 2013). All of these hallmarks occur during aging and are interconnected. Among them, cellular senescence can link to almost all of other aging hallmarks, representing a good example to study healthy aging from basic research to therapeutics (Campisi et al., 2019; Di Micco et al., 2021).

Cellular senescence is a process that leads to a state of irreversible growth arrest in response to a variety of intrinsic and extrinsic stresses (Hernandez-Segura et al., 2017). Initially, the phenomenon was found when cultured cells were shown to undergo a limited number of cell divisions *in vitro* (Hayflick and Moorhead, 1961; Hayflick, 1965). Cellular senescence is different from cell quiescence which represents a transient and reversible cell cycle arrest. Cellular senescence can be a physiologically or pathologically relevant program depending on the specific situation (Burton and Krizhanovsky, 2014; Herranz and Gil, 2018). It normally functions as a vital tumor suppressive mechanism and also plays an important role in tissue damage repair (Demaria et al., 2014; Gorgoulis et al., 2019; Moiseeva et al., 2020). However, senescent cells (SnCs) have been implicated in various age-related diseases (Palmer et al., 2015; Ogrodnik et al., 2017; Aguayo-Mazzucato et al., 2019; Zhang et al., 2019). Accordingly, selective elimination of SnCs has been exploited as a novel strategy to treat the diseases. In addition, SnCs have also been implicated in infectious diseases. For example, virus infection can induce cellular senescence, which was found to be a pathogenic trigger of cytokine escalation and organ damage, and recently found to be associated with the COVID-19 severity in the elderly (Nehme et al., 2020; Gerdes et al., 2021; Lee et al., 2021; Mohiuddin and Kasahara, 2021). Clearance of virus-induced SnCs was considered as a novel treatment option against severe acute respiratory syndrome coronavirus 2 (SARS-CoV-2) (Lee et al., 2021) and perhaps other viral infections (Camell et al., 2021).

One of the characteristics of SnCs is their ability to resist apoptosis. Until now, small molecules that can selectively kill SnCs, termed senolytics, were developed to target the proteins in the SnC anti-apoptotic pathways (SCAPs) (Childs et al., 2017; Kirkland and Tchkonia, 2017; Niedernhofer and Robbins, 2018). However, due to the high heterogeneity in gene expression and their diverse origins, SnCs may use different SCAPs to maintain their survival, making it difficult to use a single senolytic to kill all types of SnCs. Although significant progresses on the development of senolytics have been made, some proteins involved in the SCAPs have been overlooked, neither their potential of being senolytic targets have been investigated. Therefore, gaining more insights into the apoptosis-resistant mechanism of SnCs may greatly help to design or screen more effective senolytics that can be used to treat SnC-associated disorders. In this review, we systematically summarized the proteins or pathways involved in SnC apoptotic resistance documented in the publications since 1995 (Wang, 1995), when the first result of SnC resistance to apoptosis was reported.

## SnCs Are Novel Therapeutic Targets for Aging and Age-Related Diseases

Cellular senescence is the irreversible growth arrest of individual mitotic cells. It was first described by Leonard Hayflick and Paul Moorhead (Hayflick and Moorhead, 1961). In their work they found that the cultured human fibroblasts lost the ability to proliferate, reaching permanent arrest after about 50 population doublings (Hayflick and Moorhead, 1961; Hayflick, 1965). This type of cellular senescence is referred to as replicative senescence, which is caused by telomere shortening after extensive cell proliferations. Another major type of cellular senescence is stress-induced premature cellular senescence, which is resulted from exposure to various genotoxic stressors, such as oxidative stress, and chemotherapy. In addition, the activation of various oncogenes can also induce senescence termed as oncogene-induced senescence, which is caused by DNA replicative stress (Di Micco et al., 2006). After being senescent, cells display a series of cellular and molecular changes, such as enlarged cell size, irregularly shaped cell body, increased lysosomal content, accumulation of mitochondria, enlarged nuclear size and increased DNA damage (Hernandez-Segura et al., 2018). The hallmarks of cellular senescence were well summarized elsewhere (Hernandez-Segura et al., 2018). Some of them, such as increased p16 and beta-galactosidase activity, are widely used for identifying SnCs *in vitro* and *in vivo* (Childs et al., 2017).

Cellular senescence is a key mechanism for the body to prevent the propagation of damaged cells (Herranz and Gil, 2018; Gorgoulis et al., 2019). In this regards, cellular senescence acts as a tumor suppressor mechanism (Sarkisian et al., 2007; Burton and Krizhanovsky, 2014), and it also shows beneficial effects in development (Muñoz-Espín et al., 2013) and tissue repair (Demaria et al., 2014). However, SnCs can accumulate when their production persists beyond the immune clearance capacity or the immune system is compromised. Under such circumstances, SnCs may play a causative role in aging and age-related diseases by inducing reactive oxygen species (ROS) and secreting a plethora of inflammatory mediators (e.g., cytokines and chemokines), growth factors, and extracellular proteases—termed as the senescence-associated secretory phenotype (SASP) (Demaria et al., 2017; Alimirah et al., 2020). Therefore, cellular senescence is both a physiologically and pathologically relevant program depending on the specific situation.

In 2011 and 2016, Baker et al. reported that elimination of SnCs by a transgenic approach delayed the onset of several age-related diseases and prolonged lifespan in progeroid and naturally aged mice, respectively (Baker et al., 2011, 2016). They provided solid evidence for that SnCs are causally implicated in generating age-related phenotypes and that clearance of SnCs can prevent or delay tissue dysfunction and extend healthspan. Later on, accumulating data demonstrate the involvement of SnCs in various age-related diseases and disorders (Childs et al., 2015, 2017; Kirkland and Tchkonia, 2017; Niedernhofer and Robbins, 2018), such as neurodegenerative diseases (Chinta et al., 2018; Zhang et al., 2019), diabetes (Aguayo-Mazzucato et al., 2019), atherosclerosis (Childs et al., 2016), osteoarthritis (Jeon et al., 2017), and tumors (Demaria et al., 2017; Takasugi et al., 2017). In sum, these findings suggest that SnCs are an emerging therapeutic target of aging and age-related diseases.

## Targeting Senescent Cells With Senolytics and New Strategies

The evidence that elimination of SnCs by genetic strategy can extend the healthspan in mice prompts a gold rush in the development of pharmaceutical small molecules that can selectively kill SnCs to combat aging and age-related conditions. As these molecules are called ‘senolytics’, whereas those that can suppress SASP production, named senomorphics. Both senolytics and senomorphics have the potential to prevent or treat age-related diseases and to extend healthspan. However, more efforts were made on developing senolytics compared to senomorphics considering that senolytics have more favorable drug exposure, toxicity and durable effects (He et al., 2020c).

Until now, several classes of senolytics have been developed, including naturally occurring compounds and their derivatives [e.g., quercetin (Zhu et al., 2015), fisetin (Yousefzadeh et al., 2018), piperlongumine (Wang et al., 2016), EF24 (Li et al., 2019a)], cardiac glycosides [e.g., ouabain (Guerrero et al., 2019), digoxin (Triana-Martínez et al., 2019)], and targeted therapeutics [dasatinib (Zhu et al., 2015), ABT263 (Chang et al., 2016), HSP90 inhibitor (Fuhrmann-Stroissnigg et al., 2017), and FOXO4-p53 interfering peptide (Baar et al., 2017)] (Table 1). We have previously reviewed the progresses on naturally occurring and targeted senolytics (Li et al., 2019b; Ge et al., 2021). It is very encouraging that several senolytics have been approved to enter clinical trials and shown benefits as therapeutics for aging or age-related diseases (Childs et al., 2017; Kirkland and Tchkonia, 2017, 2020; Campisi et al., 2019; Thoppil and Riabowol, 2019; Di Micco et al., 2021). Even though, current senolytics still have some limitations in terms of potency, safety, specificity, broad-spectrum activity, pharmacodynamics and pharmacokinetics. For example, one of the most widely used senolytics, i.e. the combination of dasatinib and quercetin (D + Q), was well tolerated and could reduce SnC burden in patients (Thoppil and Riabowol, 2019; Kirkland and Tchkonia, 2020). However, quercetin is a polypharmacologic agent and its mechanisms of action have not been well defined. ABT263, a Bcl-2 and Bcl-xL dual inhibitor, has been extensively used as a potent and broad-spectrum senolytic agent (Chang et al., 2016). Its on-target toxicity of thrombocytopenia induced by Bcl-xL inhibition prevents its use in clinic even for tumor patients (Gandhi et al., 2011; Souers et al., 2013; Leverson et al., 2015; Ashkenazi et al., 2017). To overcome these challenges, some novel strategies, such as proteolysis-targeting chimera (PROTAC) technology (He et al., 2020b), chimeric antigen receptor (CAR) T cells (Amor et al., 2020), and β-galactosidase-modified prodrugs (Cai et al., 2020; Guerrero et al., 2020), have been developed to eliminate SnCs and shown promising therapeutic potential in the treatment of age-related diseases (Ge et al., 2021).

**TABLE 1:** Senolytic targets and agents.

Senolytic targets	Senolytic agents	References
BCL-XL/BCL-W	Navitoclax, PZ15227, A1331852, A1155463	(Zhu et al., 2015, 2017; Chang et al., 2016; Yosef et al., 2016; He et al., 2020b)
HSP90	17-DMAG	Fuhrmann-Stroissnigg et al. (2017)
MDM2	UBX0101	Jeon et al. (2017)
USP7	P5091	He et al. (2020a)
FOXO4	FOXO4-p53 interfering peptide	Baar et al. (2017)
OXR1	Piperlongumine	Zhang et al. (2018)
RTK	Dasatinib	Zhu et al. (2015)
Na^+^/K^+^ ATPase	Ouabain, Digoxin	(Guerrero et al., 2019; Triana-Martínez et al., 2019)
BRD4	JQ1, ARV825	Wakita et al. (2020)
GLS1	BPTES	Johmura et al. (2021)
FAK	R406	Cho et al. (2020)
TPP	Alkyl-diquaternary	Jana et al. (2021)
	Alkyl-monoquaternary	
LC3-II/LC3	Azithromycin, Roxithromycin	(Ozsvari et al., 2018; Yang et al., 2021)
	GL-V9	
C-IAP1/C-IAP2/BCL-2	Temozolomide	Schwarzenbach et al. (2021)
HDAC	Hinokitiol, Preussomerin C	Barrera-Vázquez et al. (2021)
	Tanshinone I	
PPARα	Fenofibrate	Nogueira-Recalde et al. (2019)

One major characteristic of SnCs is that they are resistant to apoptosis. This is attributable to the upregulation of certain proteins in the SCAPs, which enables SnCs to accumulate during aging. Most of the abovementioned senolytics were developed to selectively kill SnCs by targeting the known SCAPs. However, the transcriptional heterogeneity (Hernandez-Segura et al., 2017) and the diverse tissue origins of SnCs, render SnCs depend on different SCAPs to maintain their survival, which makes it a challenge to develop certain senolytics to eliminate SnCs in various cell types. For example, we found that ABT263 is very potent to selectively kill senescent human lung diploid fibroblasts (HDF) (Chang et al., 2016; He et al., 2020b), but it does not work in senescent HDF isolated from human foreskins (Cho et al., 2020), suggesting that HDF from different tissue origins may have different apoptotic resistance mechanisms. Therefore, gaining deep insights into the apoptosis-resistant proteins or pathways of SnCs may help to develop cell-specific or broad-spectrum senolytics to clear SnCs. In the following sections, we will focus on reviewing cell apoptosis and its significance in aging and age-related diseases, as well as summarizing the emerging proteins or pathways involved in apoptotic resistance of SnCs.

## The Significance of Apoptosis in Aging and Age-Related Diseases

Apoptosis is defined as a programmed cell death, which has received a lot of attention because of its importance during tissue development (Wanner et al., 2020), and maintenance of tissue homeostasis (Elmore, 2007; Günther et al., 2013; Kile, 2014; Singh et al., 2019). Apoptosis occurs when cells undergo exogenous stimuli or intrinsic signals and is required to maintain the integrity and homeostasis of tissues, and dysregulation of which is implicated in the development of various diseases (Singh et al., 2019). For example, increased cellular apoptosis is known to contribute to age-related diseases in the brain (Mattson et al., 2001). Unlike cellular necrosis, a way of cell death resulting from acute cellular injury, apoptosis does not induce an inflammation response to cause a bystander effect.

**FIGURE 1:**
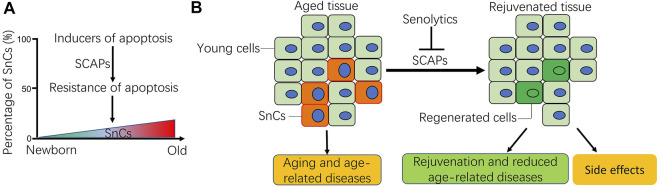
Schematic of apoptotic resistance of senescent cells (SnCs) in aging and age-related diseases. **(A)** During the aging process, aging-related disruptions in systemic and inter-cell signaling, together with cell-autonomous damage and mitochondrial malfunction result in either increased or decreased cell apoptosis depending on cellular context (Tower, 2015). As for SnCs, their percentage usually increases in tissues with age as they acquire resistance to apoptotic stimuli via the SnC anti-apoptotic pathways (SCAPs). **(B)** Accumulation of SnCs in tissues contributes to aging and the occurrence of age-related diseases. Senolytics can selectively clear the SnCs by targeting the SCAPs, which promote the regeneration of young cells, rejuvenate aged tissues and reduce age-related diseases. Sometimes, senolytics can also cause side effects, more caution should be exercised for the systemic use of senolytics for health benefits. Note: **B** was modified from our previous publication (Li et al., 2019a; Li et al., 2019b).

Aging has been associated with decreased apoptosis in some cell types (Figure 1A), such as adipose mesenchymal stem cells (Alt et al., 2012), and bone marrow mesenchymal stem cells (Wilson et al., 2010). *In vivo*, it has been reported that apoptotic potential was dramatically reduced in the liver of aged rats challenged with a DNA-damaging agent (Suh et al., 2002). Similarly, apoptosis induced by radiation was significantly reduced in peripheral blood lymphocytes isolated from aged mice (Polyak et al., 1997). In humans, the peripheral blood mononuclear cells (PBMCs) from old people and centenarians showed an increased resistance to apoptosis induced by 2-deoxy-D-ribose (dRib), which may contribute to immunosenescence (Monti et al., 2000). Consistently, there is a reduction in the markers of apoptosis in human serum during aging (Kavathia et al., 2009). In a few cases, the apoptosis is increased in some cell types during aging. For example, there is a significant loss of cells in the thymus and bone marrow, associated with an increase in the number of apoptotic lymphocytes during the aging process (Sainz et al., 2003). The discrepancy of apoptosis in different cell types during aging is likely due to the age-related disruptions in systemic and intracellular signaling combined with cell-autonomous effects (Tower, 2015). Because apoptosis is required for normal cell turnover and tissue homeostasis, dysregulation of apoptosis at old age is increasingly implicated in various age-related diseases, such as tumor (DePinho, 2000), and neurodegenerative diseases (Erekat, 2021). However, SnCs are highly resistant to apoptosis (Wang, 1995), likely enabling them accumulate during aging (Figure 1A), which acts as an important contributor to aging (Salminen et al., 2011), and age-related diseases (Kirkland and Tchkonia, 2017; Niedernhofer and Robbins, 2018; Thoppil and Riabowol, 2019; Di Micco et al., 2021; Ge et al., 2021). Thus, it is imperative to understand why SnCs are resistant to apoptosis. A better understanding of the underlying mechanisms for apoptotic resistance of SnCs is likely to provide novel molecular targets for the development of therapeutic senolytics to combat aging and age-related diseases (Figure 1B).

## Apoptotic Resistance and the Underlying Mechanism in SnCs

Since the first observation of apoptotic resistance in SnC human fibroblasts was reported in 1995 (Wang, 1995), the phenomenon was later found in a variety of cell types when they became senescent, including senescent immune cells (Spaulding et al., 1999), keratinocytes (KCs) (Chaturvedi et al., 2004), human endometrium-derived mesenchymal stem cells (MESCs) (Burova et al., 2013) and hepatic stellate cells (HSC) (Kawada, 2006). There are various mechanisms that promote the survival of SnCs *in vitro* and *in vivo*, such as metabolic reprogramming, activation of unfolded protein response, and immune evasion (Soto-Gamez et al., 2019). In this review, we focus on the SCAPs, as most of the known senolytics were developed by targeting individual SCAPs (Figure 1B). Emerging SCAPs are reported along with the findings of SnC resistance to apoptosis in different cell types, providing new target candidates for developing either cell-specific or broad-spectrum senolytics. In this section, we review the findings of SnC apoptotic resistance reported since 1995 and tempt to broaden the knowledge of SCAPs for the development of senolytics. The known proteins or pathways responsible for apoptotic resistance in SnCs are listed in Table 2.

**TABLE 2:** Proteins implicated in the apoptotic resistance of senescent cells (SnCs).

Proteins	Cell type	Apoptotic stimuli	Type of Senescence	References
Bcl-2 family	Human lung fibroblast WI-38	Serum deprivation	Replicative senescence	Wang, (1995)
	Human diploid fibroblast from foreskin	H_2_O_2_, staurosporine, thapsigargin	Replicative senescence	Ryu et al. (2007)
	Human CD8^+^ T cells	Anti-CD3 mAb, anti-Fas mAb, IL-2 withdrawal, staurosporine, galectin-1, dexamethasone, mild heat shock	Replicative senescence	Spaulding et al. (1999)
	Human diploid fibroblast IMR-90	H_2_O_2_	Replicative senescence	Sanders et al. (2013)
	Human fibroblast WI-38, IMR–90 and human renal epithelial cells	ABT263	IR induced senescence	Chang et al. (2016)
	IMR-90, MEF	TNFα+cycloheximide, UV	Etoposide induced senescence; Replicative senescence; Oncogene-induced senescence	Yosef et al. (2016)
	Cholangiocyte	TRAIL	LPS induced senescence	O’Hara et al. (2019)
P53	Human lung fibroblast WI-38	Actinomycin D, UV, etoposide, cisplatin	Replicative senescence	Seluanov et al. (2001)
	Foreskin derived fibroblast HCA2 and IMR-90	NA	Replicative senescence	Jackson and Pereira-Smith, (2006)
	Human skin fibroblast	UV	UV induced senescence	Chen et al. (2008)
	Keratinocyte isolated from neonatal foreskin	UV	IFN plus TPA induced senescence	Chaturvedi et al. (2004)
Heat shock protein	MEF	NA	Primary MEFs from *Ercc1* ^*−/−*^ mice	Fuhrmann-Stroissnigg et al. (2017)
MAPK-NF-κB	Keratinocyte isolated from neonatal foreskin	UV	Replicative senescence	Chaturvedi et al. (1999)
	Human primary foreskin fibroblast	UV	Replicative senescence; H_2_O_2_ induced senescence	Yeo et al. (2000)
	Human diploid fibroblast from foreskin	H_2_O_2_, staurosporine, thapsigargin	Replicative senescence	Kim et al. (2011)
Insulin/IGF axis	Human diploid fibroblast from foreskin	Serum deprivation	Replicative senescence	Hampel et al. (2005)
Caspase-3	Human lung fibroblast WI-38	UV, staurosporine	Replicative senescence	Marcotte et al. (2004)
Survivin	Normal human skin fibroblast (HFSN1) and mouse embryonic fibroblast (MEF)	γ-ray, Cisplatin, H_2_O_2_, UV	Replicative senescence	Al-Khalaf and Aboussekhra, (2013)
	HeLa and HCT116	Doxorubicin	Doxorubicin induced senescence	Ma et al. (2016)
Gelsolin, FAK and MVP				
Gelsolin	Human diploid fibroblast from foreskin	Menadione	Replicative senescence	(Ahn et al., 2003a; 2003b)
FAK	Human diploid fibroblast from foreskin	H_2_O_2_, staurosporine	Replicative senescence	Ryu et al. (2006)
MVP	Human diploid fibroblast from foreskin	H_2_O_2_, staurosporine, thapsigargin	Replicative senescence	(Ryu et al., 2008; Ryu and Park, 2009)
Others				
DcR2	Human diploid fibroblast IMR-90 and primary human hepatic myofibroblasts (activated HSCs)	NA	Etoposide	Sagiv et al. (2013)
GLS1	hHCA2, hIMR-90, hRPE-1, and mouse embryo fibroblast (MEF)	NA	Nutlin3a, Doxorubicin, tert-butyl hydroperoxide, Replicative senescence	Johmura et al. (2021)
SENEX	The human DLBCL (Diffuse large B-cell lymphoma) cell line OCI-LY8	Doxorubicin	Doxorubicin induced senescence	Wang et al. (2020)
NA	Primary foreskin fibroblast	Ceramide	Replicative senescence	Hampel et al. (2004)
NA	Human mesenchymal stem cells (hMESCs)	H_2_O_2_	H_2_O_2_ induced senescence	Burova et al. (2013)
NA	Cells in aged liver	Methyl methanesulfonate	Naturally aged rats	Suh et al. (2002)

NA, not available.

### The Bcl-2 Family Members

The Bcl-2 family proteins, consisting of antiapoptotic and proapoptotic proteins, play critical roles in the regulation of apoptosis (Youle and Strasser, 2008). The Bcl-2 antiapoptotic proteins are multi-BH-domain proteins including Bcl-2, Bcl-xL, Mcl-1, Bcl-w and Bfl1. They can inhibit apoptosis by binding to the multi-BH-domain and BH3-only proapoptotic proteins. Wang et al. first observed that aged human WI-38 cells were resistant to programmed cell death induced by serum deprivation. They found that the levels of Bcl-2 was undetectable in young WI-38 cells, but remained unchanged in senescent WI-38 cells after serum deprivation (Wang, 1995). This finding suggests that Bcl-2 might be primarily responsible for the resistance of cell death in senescent WI-38 cells. In HDF, several apoptotic stimuli such as H_2_O_2_, staurosporine and thapsigargin, can induce the downregulation of Bcl-2 and apoptosis in young cells but not in SnCs (Ryu et al., 2007). Spaulding et al. reported that replicative senescence in CD8^+^ T cells was associated with a significant increase in resistance to apoptosis, which was also associated with increased Bcl-2 expression (Spaulding et al., 1999). On the contrary, Bcl-2 was reduced in senescent HSC that were sensitive to TNF-α-induced cell death (Novo et al., 2006). Mechanistically, global and locus-specific histone modifications of chromatin regulate the gene expression of *Bcl-2* and *Bax* in senescent fibroblasts, which in turn may mediate the resistance of apoptosis in SnCs (Sanders et al., 2013).

In contrast, Sasaki et al. observed the apoptotic resistance in several cell lines (human foreskin fibroblast strain HCA2, human fetal lung fibroblast strains TIG-1, TIG-3 and WI-38) when they became senescent (Sasaki et al., 2001). However, in these SnCs the Bcl-2 family members were not responsible for the resistance to apoptosis. Instead, they attributed the resistance to cell cycle arrest (Sasaki et al., 2001). Different from above findings, we and others demonstrated that Bcl-xL is primarily responsible for SnC resistance to apoptosis as inhibition of Bcl-xL with a Bcl-xL specific inhibitor (such as A-1331852 and A1155463) (Zhu et al., 2017) or a Bcl-2 and Bcl-xL dual inhibitor (such as ABT263 and ABT737) can effectively induce apoptosis in a variety of SnCs (Rochette and Brash, 2008; Chang et al., 2016; Yosef et al., 2016; Zhu et al., 2016; Baar et al., 2017; Zhu et al., 2017; He et al., 2020b), whereas inhibition of Bcl-2 alone with its specific inhibitor has no or weak effect on SnC survival (Chang et al., 2016; Yosef et al., 2016; He et al., 2020b). The induction of Bcl-xL in SnC and its role in mediating apoptotic resistance is likely regulated by the transcription factor ETS protooncogene 1 (ETS1) (O’Hara et al., 2019). Although ABT263 is one of the most potent and broad-spectrum senolytics identified to date (Zhu et al., 2015, 2017; Chang et al., 2016), Bcl-xL inhibition with ABT263 or other small molecular inhibitors induces platelet apoptosis and results in severe thrombocytopenia, which prevents their use in the clinic—even for tumor patients (Gandhi et al., 2011; Souers et al., 2013; Leverson et al., 2015; Ashkenazi et al., 2017). We have successfully used the PROTAC technology to overcome the on-target drug toxicity, and improved the senolytic activity of ABT263 (He et al., 2020b). As one of the most important Bcl-2 family members, Mcl-1 plays critical roles in suppressing apoptosis in tumor cells (Ramsey et al., 2018; Mukherjee et al., 2020; Seiller et al., 2020), which however may not affect the survival of SnCs, as inhibition of Mcl-1 with its specific inhibitor did not cause cell death in SnCs (Chang et al., 2016; He et al., 2020b). Of note is that Mcl-1 expression varies in SnCs induced by different stress-stimulus (Yosef et al., 2016), and it is a highly regulated protein with a short half life (Michels et al., 2005), implying that Mcl-1 may circumvent the inhibition of Bcl-2 or Bcl-xL and help SnCs to acquire apoptotic resistance. In sum, Bcl-2 family members are implicated in the apoptotic resistance of SnCs, but their expression levels and roles differ depending on cell types and senescence inducers.

### The p53 Pathway

p53 is a well-known tumor suppressor that acts as a double-edged sword in the regulation of cellular senescence, aging, and tumor (Johmura and Nakanishi, 2016; Wu and Prives, 2018). The host defense function of p53 in protecting tissues against tumor growth via induction of apoptosis and cellular senescence has been widely studied in tumors. The p53 levels and activities increase when cells enter a presenescent state upon activation of the DNA damage response (DDR), triggering the initiation of cellular senescence (Hernandez-Segura et al., 2018). p53 can promote apoptosis through transcription-dependent and -independent mechanisms (Fridman and Lowe, 2003). The transcriptional pathway involves the induction of pro-apoptotic Bcl-2 family members (i.e., Bax, Bid, Noxa, and Puma), and repression of anti-apoptotic proteins (i.e., Bcl-2, Bcl-xL, and survivin). The non-transcriptional regulation of apoptosis by p53 includes its direct interaction with members of the Bcl-2 family anti-apoptotic proteins to control mitochondrial outer membrane permeabilization (MOMP) (Fridman and Lowe, 2003).

The role of p53 in regulating apoptotic resistance in SnCs has been reported in multiple cell types. Seluanov et al. studied the responses of cultured young and old WI-38 to a variety of genotoxic stresses and found that young cells were able to undergo p53-dependent and p53-independent apoptosis. In contrast, senescent fibroblasts were unable to undergo p53-dependent apoptosis. They concluded that stabilization of p53 in response to DNA damage is impaired in old fibroblasts, resulting in the resistance to apoptotic stimuli (Seluanov et al., 2001). Gansauge et al. also observed the same phenomenon in the same cell line (Gansauge et al., 1997). Similar findings were reported in senescent KCs (Chaturvedi et al., 2004), human skin fibroblasts (Chen et al., 2008) and HCA2 cells (Jackson and Pereira-Smith, 2006). The cellular levels of p53 usually increase in response to DNA damage stimuli. However, the increased levels of p53 subside when cells become fully senescent. This phenomenon was observed in senescent KCs (Kim et al., 2015), human fibroblast cell lines (Sisoula et al., 2011; Johmura and Nakanishi, 2016), human prostate epithelial and uroepithelial cells (Schwarze et al., 2001). The downregulation of p53 levels in SnCs may be attributable to the upregulation of C-terminus of Hsp70-interacting protein (CHIP) (Sisoula et al., 2011) and SCFFbxo22 (Johmura et al., 2016) E3 ligases, which promote p53 degradation through the ubiquitination-proteasome system. The reduction of p53 in SnCs may protect them from apoptosis and cause the accumulation of SnCs and higher prevalence of tumor during aging, which agrees with the finding that p53 activity was reduced along with accumulation of SnCs in aged tissues (Feng et al., 2007; Baker et al., 2016). Therefore, restoration of p53 activity has the potential to eliminate SnCs by inducing apoptosis. Baar et al. designed a peptide (termed as proxofim peptide) comprising part of the p53-interaction domain in FOXO4, which selectively induced p53 nuclear exclusion and cell-intrinsic apoptosis in SnCs (Baar et al., 2017; Zhang et al., 2020). However, there remain challenges using a peptide as a therapeutic. Inhibiting the interaction between MDM2 and p53 can also increase p53 stability and activity (Moll and Petrenko, 2003). UBX0101, an inhibitor of MDM2, has been shown to selectively kill SnCs in culture and treat post-traumatic osteoarthritis *in vivo* (Jeon et al., 2017). Nevertheless, UBX0101 was failed for the treatment of osteoarthritis in patients in a phase II clinical trial (Lane et al., 2021). We have previously reported that the ubiquitin-specific peptidase 7 (USP7) may be a novel target for senolysis as inhibition of USP7 can selectively induce apoptosis of SnCs by increasing the level of p53 (He et al., 2020a). However, USP7 is widely expressed in many tissues and has multiple substrates with different physiological functions (Zhou et al., 2018; Pozhidaeva and Bezsonova, 2019). It has yet to be determined whether USP7 can be safely used to clear SnCs without causing significant side effects.

### Heat Shock Proteins

Heat shock proteins (HSPs) are evolutionarily conserved proteins whose expression is induced by different kinds of stresses (Jäättelä, 1999; Jego et al., 2013). They are involved in protein folding and maturation of various proteins and play an important role in regulation of cellular response to homeostatic challenges (Chatterjee and Burns, 2017). HSPs regulate protein assembly, secretion, transportation, translocation, and protein degradation. According to their molecular sizes, HSPs are grouped into six families, including HSP27, HSP40, HSP60, HSP70, HSP90 families, and the family of large HSPs (HSP110 and glucose-regulated protein 170, GRP170) (Chatterjee and Burns, 2017). When cells are subjected to stress, they can undergo either senescence or cell death and HSPs are involved in both two responses (Takayama et al., 2003; He et al., 2019; Srivastava et al., 2019; Kanugovi et al., 2020; Omer et al., 2020; Kanugovi Vijayavittal and Amere Subbarao, 2021). The role of HSPs in regulating cell apoptosis has been well reviewed elsewhere (Takayama et al., 2003; Lanneau et al., 2008). As the expression/activity of HSPs is significantly higher in tumors and responsive to different death stimuli (Jego et al., 2013), inhibition of HSPs has emerged as a novel therapeutic strategy for tumor therapy (Chatterjee and Burns, 2017).

Among the HSP families, HSP90 has been implicated as an anti-apoptotic and pro-survival factor in SnCs, and the inhibitor of HSP90 was identified as a new class of senolytic (Fuhrmann-Stroissnigg et al., 2017, 2018). Inhibition of HSP90 by its inhibitors, such as 17-DMAG, can sufficiently reduce the level of phosphorylated AKT and selectively induce apoptosis in SnCs (Fuhrmann-Stroissnigg et al., 2017, 2018). However, most of the known HSP90 inhibitors may have limited usage as senolytics because they can cause some dose-limiting toxicities and have poor pharmacokinetic profiles (Sanchez et al., 2020). These limitations may preclude their clinical use as an anti-aging agent as older people are more susceptible to adverse drug effects than younger individuals (He et al., 2020c).

### The Autophagy-Lysosomal Pathway

Autophagy is an evolutionarily conserved process in eukaryotic cells, which is involved in scavenging and recycling senescent or damaged organelles and biological macromolecules by lysosomal degradation to maintain cellular homeostasis. Autophagy can be a physiologically or pathologically relevant program depending on the specific situation. In normal situations, autophagy can be induced by physiological signals such as starvation, to facilitate cell survival (Giampieri et al., 2019). Caloric restriction (CR) is one of the most important inducers of autophagy, leading to an increase of lifespan and delaying the onset of age-related diseases (Bergamini et al., 2007). Nevertheless, excessive autophagy is detrimental and results in “autophagic cell death” that describes a form of programmed cell death morphologically distinct from apoptosis. In autophagic programmed cell death, there is early degradation of organelles but preservation of cytoskeletal elements until late stages (Levine and Yuan, 2005). Different from necrosis, both apoptotic and autophagic cell death are characterized by the lack of a tissue inflammatory response. Autophagy have been linked to aging and various pathological conditions (Green et al., 2011; Giampieri et al., 2019), and thus it has become a major target for drug discovery and development (Kroemer, 2015; Leidal et al., 2018). However, as autophagy can act as both a cell survival and death mechanism, it is challenging to selectively turn on or turn off autophagic survival and death pathways in the treatment of autophagy-related diseases (Levine and Yuan, 2005). Beyond the abovementioned roles, autophagy has been shown to influence cellular immune responses (Ma et al., 2013). Particularly, autophagy can influence the antigenic profile of antigen-donor cells (ADCs) and their ability to release immunogenic signals (Michaud et al., 2011), as well as the survival, differentiation, and function of antigen-presenting cells (APCs) and T lymphocytes (Pua et al., 2007; Jia and He, 2011; Wildenberg et al., 2012; Fiegl et al., 2013). Autophagic responses in ADCs can enhance the release of “find-me” and “eat me” signals, which attract APC progenitors and facilitate antigen uptake, respectively (Ma et al., 2013). Theoretically, the autophagy-enhanced cellular immune responses may facilitate the immune system to recognize and eliminate SnCs, which has yet to be validated. The relationship between autophagy and cellular senescence is inconclusive (Gewirtz, 2013). Some studies suggest that autophagy is activated during senescence and inhibition of autophagy delays the senescence phenotype (Young et al., 2009; Gewirtz, 2013; Zhang et al., 2017), whereas increasing evidence reveals a negative correlation between them (Kang et al., 2011; Tai et al., 2017). For example, rapamycin, a mammalian target of rapamycin complex 1 (mTOR1) inhibitor and an autophagy activator, can efficiently suppress cellular senescence (Demidenko et al., 2009; Tai et al., 2017), and extend the life span of mice (Harrison et al., 2009). Fenofibrate (FN), a PPARα agonist used for dyslipidemia in humans, was reported to protect against cartilage degradation by reducing the number of senescent chondrocytes via inducing apoptosis in combination with increasing autophagic flux (Nogueira-Recalde et al., 2019). In addition, a BET family protein degrader was shown to provoke senolysis by targeting the attenuation of nonhomologous end joining (NHEJ) and autophagy in SnCs (Wakita et al., 2020). Lysosomes are degradative organelles essential for cell homeostasis that regulate various biological processes (Gómez-Sintes et al., 2016). Senescence-associated beta-galactosidase (SA-beta-gal) is the most common marker of lysosomal activity and one of the first tests used to determine senescence (Lee et al., 2006). Enhanced lysosomal activity can protect cells from oxidative stresses (Chakraborty et al., 2019; Nagakannan et al., 2020; Li et al., 2021), likely contributing to the resistance of apoptosis in SnCs. However, different forms of stress can induce lysosomal membrane permeabilization (LMP), leading to the translocation to the cytoplasm of intralysosomal components, such as cathepsins, inducing lysosomal-dependent cell death (LDCD) (Wang et al., 2018). Accumulating evidence indicate that aging significantly influences lysosomal activity by altering the physical and chemical properties of lysosomes (Gómez-Sintes et al., 2016). Most cells display an age-associated increase in lysosome number and size. The intracellular pH in SnCs was lowered by lysosomal membrane damage, but SnCs can resist the lowered pH by producing ammonia *via* glutaminolysis, which can neutralize the lower pH (Johmura et al., 2021). On the contrary, it was reported that lysosomes exhibited higher pH in aged animals than young mice (Liu et al., 2008). Lysosomal reacidification by inhibiting the ataxia telangiectasia mutated (ATM) induced functional recovery of the lysosome, which led to alleviated cellular senescence by accelerating the removal of dysfunctional mitochondria and recovering the mitochondrial function (Kang et al., 2017). Taken together, the autophagy-lysosomal pathway plays critical roles in maintaining cellular homeostasis, targeting the proteins in the pathway have become an attractive anti-aging strategy by either suppressing senescence or inducing apoptosis of SnCs.

### Epigenetic Regulation

Alterations in the methylation of DNA or post-translational modification of histones, and other chromatin-associated proteins, can induce epigenetic changes that contribute to the aging process (López-Otín et al., 2013). We and others have previously reviewed the effects of DNA methylation on aging and longevity (Xiao et al., 2016; Sidler et al., 2017; Kane and Sinclair, 2019; Morris et al., 2019). Likewise, epigenetic changes have significant impact on the senescence phenotypes, notably the proliferative arrest and SASP (Nacarelli et al., 2017). For example, the INK4-ARF locus encodes proteins that drive cell growth arrest, and the polycomb group (PcG) proteins can epigenetically regulate the INK4-ARF locus, as well as catalyze histone modifications that promote changes in chromatin structure, leading to transcriptional repression (Simboeck et al., 2011). SIRT1, an NAD^+^-dependent protein deacetylase, suppresses the SASP through epigenetic gene regulation (Hayakawa et al., 2015; Hekmatimoghaddam et al., 2017). Besides the impacts on cellular senescence and SASP, epigenetic mechanisms are involved in the apoptotic resistance in SnCs. CpG nucleotide-rich regions in mammalian genomes are called CpG islands, which reside in very close proximity to gene promoters (Fatemi et al., 2005). In tumor cells, CpG islands at proapoptotic gene promoters are mostly hypermethylated due to methyltransferase (DNMT) overexpression (Roll et al., 2008). Hypermethylation blocks both intrinsic and extrinsic apoptosis by modulating the expression of major players of cell death cascade, as previously reviewed elsewhere (Elmallah and Micheau, 2019; Ozyerli-Goknar and Bagci-Onder, 2021). However, the expression of DNMT was found to be downregulated in SnCs although they harbour some features of the tumor epigenome (Cruickshanks et al., 2013). Thus, whether DNA methylation contributes to the apoptotic resistance in SnCs has yet to be investigated. In contrast, there is evidence showing that histone modification may affect the sensitivity of SnCs to cell death stimuli. Sanders et al. revealed that both global and locus-specific histone modifications of chromatin regulated altered Bcl-2:Bax gene expression in senescent fibroblasts, contributing to its apoptosis-resistant phenotype (Sanders et al., 2013). The heterochromatinization that surrounds and borders double-strand breaks (DSBs) enhances the pro-survival responses in SnCs, as evidenced by that HDAC inhibition triggered apoptosis (Di Micco et al., 2006; Paluvai et al., 2020). Panobinostat, an FDA-approved histone deacetylase inhibitor, was found to be a post-chemotherapy senolytic agent with the potential to kill persistent SnCs in non-small cell lung cancer (NSCLC) and head and neck squamous cell carcinoma (HNSCC) (Samaraweera et al., 2017). However, our preliminary data show that panobinostat was toxic to normal cells (data now shown). Therefore, whether the histone deacetylase inhibitors can be anti-aging senolytic targets has yet to be investigated.

### The MAPK-NF-κB Axis

The nuclear factor κB (NF-κB) is a transcription factor complex consisting of homo- and heterodimers of five members of the Rel family including RelA (p65), RelB, c-Rel, NF-κB1 (p50/p105), and NF-κB2 (p52/p100). The NF-κB pathway transcriptionally controls a large set of target genes that play important roles in cell survival, inflammation, and immune responses (Hayden and Ghosh, 2008). Previous studies have correlated NF-κB signaling with cellular senescence. For example, indoxyl sulfate can induce cellular senescence via activation of the ROS-NF-κB-p53 pathway in proximal tubular cells (Shimizu et al., 2011). It has been well known that various DNA damage stimuli can activate NF-κB signaling, which stimulates the production of SASP in SnCs (Salminen et al., 2012), and regulates expression of genes that regulate apoptosis, cell cycle progression, and inflammation. Recently, we reported that SnCs not only expressed higher basal levels of various inflammatory cytokines and chemokines as a manifestation of the SASP, but also exhibited hyper-activation for the induction of a variety of inflammatory mediators in response to LPS, IL1β and TNFα stimulation (Budamagunta et al., 2021). This senescence-associated hyper-activation is mediated in part via the NF-κB pathway (Budamagunta et al., 2021). More importantly, NF-κB plays a crucial role in the induction of tumor resistance to apoptosis (Mortezaee et al., 2019). We previously reported that the curcumin analog EF24, a new senolytic which can downregulate anti-apoptotic family proteins, likely via the proteasome degradation of the Bcl-2 as the proteasome inhibitor MG132 partially prevented the degradation effect of EF24 (Li et al., 2019a). EF24 is also an anti-tumor agent that can kill melanoma cells effectively via downregulating the expression of Bcl-2 and IAP by inhibiting the NF-κB signaling (He et al., 2021). In an early study, senescent KCs were found to be resistant to apoptosis, which was associated with the anti-apoptotic role of NF-κB (Chaturvedi et al., 1999). NF-kB is upregulated in a variety of tissues with aging, and the inhibition of NF-kB has been shown to delay the onset of aging-related symptoms and pathologies such as diabetes, atherosclerosis, and tumor (Kanigur Sultuybek et al., 2019). Metformin is an anti-diabetic medication in type 2 diabetes (T2DM) (Dunn and Peters, 1995), and it has become a therapeutic candidate in the improvement of ageing and aging-related diseases (Hu et al., 2021). One of the major roles of metformin is to inhibit the NF-κB pathway, by which it reduce the susceptibility to age-related diseases (Kanigur Sultuybek et al., 2019). Many naturally-occurring compounds were reported to have anti-aging effects, either via triggering the apoptosis of SnCs (such as quercetin, fisetin, piperlongumine) (Li et al., 2019b), or inhibiting production of SASP via suppressing the NF-κB pathway (such as apigenin) (Lim et al., 2015; Perrott et al., 2017). In addition, some small chemical inhibitors of NF-κB, such as BAY 11-7082 (Pierce et al., 1997), also show good activity in reducing the expression of inflammatory cytokines (Lappas et al., 2005; Juliana et al., 2010; Zang et al., 2017), and protecting organs from oxidative injury (Kim et al., 2010; Kolati et al., 2015). However, more deep studies are needed to test whether NF-κB inhibitors could be safe anti-aging agents in the future.

The mitogen-activated protein kinase (MAPK) family can act as an upstream of the NF-κB pathway. MAP kinases are grouped into three families: stress-activated protein kinases (p38/SAPKs), Jun amino-terminal kinases (JNKs), and extracellular-signal-regulated kinases (ERKs). In cellular senescence, p38 induces the expression of the SASP largely by increasing NF-κB transcriptional activity (Freund et al., 2011; Budamagunta et al., 2021). JNKs play important roles in acquiring the resistance of senescent human fibroblasts to UV-induced DNA fragmentation (Yeo et al., 2000). In addition, ERK and p38 are also involved in the apoptotic resistance in SnCs (Kim et al., 2011). Kim et al. revealed that the nuclear translocation of activated ERK or p38 was implicated in the transduction of death signals, and that a decrease in nucleocytoplasmic trafficking of these proteins in SnCs caused the senescence-associated resistance to apoptosis (Kim et al., 2011). In conclusion, the MAPK-NF-κB axis plays important roles in mediating apoptotic resistance as well as modulating the production of various inflammatory cytokines in SnCs.

### The Insulin/IGF Axis

The insulin/IGF signaling pathway plays a major role in determining the rate of aging in many species (Gems and Partridge, 2001; Cheng et al., 2005), which is an evolutionarily conserved mechanism from yeast to humans (Barbieri et al., 2003). In higher eukaryotes, signal transduction through the IGF pathway is modulated by IGF-binding protein (IGFBPs) family, among which IGFBP-3 regulates proliferation and survival in many mammalian cell types (Firth and Baxter, 2002). Moreover, another IGF protein IGFBP-7 can also induce cellular senescence and apoptosis through autocrine/paracrine pathways in melanocytes (Wajapeyee et al., 2008). However, why SnCs secret IGF proteins but can resist their pro-apoptotic effect is not known at the present. Hampel et al. found that IGFBP-3 accumulated in conditioned medium of senescent human fibroblasts, which may contribute to growth arrest of these cells. As IGFBP-3 can enhance apoptosis by activating intracellular regulators of apoptosis, whereas IGFBP-3 cannot be endocytosed by SnCs, which may contribute to the well-established apoptotic resistance of senescent human fibroblasts (Hampel et al., 2005). Further we also have previously reported that IGFBP-3 gene polymorphism rs11977526 with longevity in Chinese (He et al., 2014). These findings suggest that the insulin/IGF axis is an important determinant of aging, and acts as a mechanism by which SnCs acquire resistance to apoptosis.

### Caspase-3

Caspases are a family of endoproteases that provide critical links in cell regulatory networks controlling inflammation and cell death. They are inactive until being cleaved at specific aspartate residues (McIlwain et al., 2013). Wang’s group reported the first observation of apoptotic resistance in senescent fibroblasts in 1995 (Wang, 1995). A few years later, they found that caspase-3 gene expression level was decreased in human senescent fibroblasts (WI-38) and could not be activated by UV or staurosporine treatment. They concluded that the resistance to apoptotic death seen in senescent fibroblasts was not simply due to increased Bcl-2 levels, but also attributable to a lack of caspase activity (Marcotte et al., 2004). However, in some cell types, cell death seems to be independent of caspase activity, such as in HDF isolated from skin, given that the treatment of HDF did not trigger the cleavage of caspase-3 or poly (ADP-ribose) polymerase (PARP) (Ryu et al., 2006).

### Survivin

Survivin is an important anti-apoptotic protein. It is highly expressed in most tumors, which is also generally arised in cells of older individuals (Unruhe et al., 2016). Al-Khalaf et al. found that survivin and phospho-survivin were accumulated in aged normal human skin fibroblasts and mice organs, which may be attributable to HSP90-mediated stabilization of survivin. Inhibition of survivin by flavopiridol or shRNAs increased the apoptotic response of old fibroblasts to various genotoxic agents by restoring the pro-apoptotic Bax/Bcl-2 ratio and increasing the levels of cleaved caspase-3 and PARP (Al-Khalaf and Aboussekhra, 2013). Another study indicated that nuclear accumulation of Yes-associated protein (YAP) could promote the survival of senescent tumor cells by increasing the expression of survivin (Ma et al., 2016). Whether survivin can be a significant senolytic target has yet to determined.

### Gelsolin, FAK and MVP

Park’s group showed that the protein and mRNA levels of gelsolin, a Ca^2+^-dependent actin regulatory protein, were increased in senescent HDF. Downregulation of gelsolin in senescent HDF resulted in increased sensitivity to menadione-induced apoptotic cell death (Ahn et al., 2003b; 2003a). The effect of gelsolin in mediating apoptotic resistance of SnCs was independent of Bcl-2 (Ahn et al., 2003a). Later, they reported that the focal adhesion kinase (FAK) might differently regulate apoptosis and focal adhesion formation in SnCs (Ryu et al., 2006). Furthermore, they identified a novel senolytic drug R406 that showed selective toxicity in HDF by inhibiting FAK and p38MAPK activity (Cho et al., 2020). They also found that the major vault protein (MVP) was markedly increased in senescent HDF as well as in aged organs, downregulation of MVP increased the sensitivity of senescent HDF to apoptosis by modulation of Bcl-2 expression (Ryu et al., 2008). MVP was a transcriptional target of p53 (An et al., 2009), being a potential therapeutic target for modulation of resistance to apoptosis (Ryu and Park, 2009). These new proteins involved in resistance to apoptosis expanded the scope of existed SCAPs, which have great potential to be senolytic targets.

### The Extrinsic Apoptosis Pathway

The extrinsic apoptosis pathway is triggered by the binding of death ligands to their appropriate death receptors (DRs) on the cell surface (Sayers, 2011). The best-described death ligands belong to the TNF family of proteins, comprising TNF, FasL, and TRAIL, which are predominantly produced by immune cells such as T cells, NK cells, NKT cells, macrophages, and dendritic cells. The DRs include TNF receptor 1 (TNFR1), CD95 (Fas), and TNF-related apoptosis inducing ligand-receptor (Ashkenazi, 2008; Sayers, 2011; Nair et al., 2014). Upon ligand binding, DRs trimerizes and recruits specialized adaptor proteins via their death domain (DD), such as Fas-associated death domain (FADD), which transmits the death signal from the cell surface to the intracellular signaling pathways. In turn, DD can recruit pro-caspase-8 and form a death-inducing signaling complex (DISC) capable of activating inducer caspases‐8, which are then processed into its mature form. The mature caspases-8 subsequently activates downstream effector caspases such as caspase-3, and trigger apoptosis. DRs-mediated apoptosis can be inhibited by a protein called c-FLIP which can bind to DD and caspase-8, rendering them ineffective (French and Tschopp, 1999). In addition, decoy receptors (DcRs) have similar extracellular structure to DRs but lack an intracellular death domain, which can bind the ligand but cannot transmit death signals (Ashkenazi, 2002). Therefore, both c-FLIP and DcRs act as inhibitory mechanisms of extrinsic apoptosis. The expression of c-FLIP was reported to decline with age in normal thymus, and negatively associated with the levels of senescence markers in primary thymic epithelial cells, suggesting that cFLIP may not play a role in the apoptotic resistance of SnCs (Belharazem et al., 2017). Nevertheless, the expression of cFLIP was downregulated by senescence-secreted SASP via an activation of the Myc oncogene, which can sensitize pretransformed cells to TRAIL-induced apoptosis (Vjetrovic et al., 2014). These findings suggest that SnCs may not depend on cFLIP for survival, instead they may regulate cFLIP to prime pretransformed cells to undergo apoptosis. DcR2 (TNFRSF10D) have been shown to be upregulated in SnCs (Sagiv et al., 2013), which represents as a marker of cellular senescence (Chen et al., 2017). DcR2 may in part account for the resistance of SnCs to apoptosis, as suggested by that TRAIL induced twofold more killing in senescent IMR-90 cells with DcR2 silencing compared with the control (Sagiv et al., 2013). Similarly, DcR1 (TNFRSF10C) was also observed to be upregulated in SnCs and was viewed as a senescence marker (Shah et al., 2013; Wiley et al., 2017). As DcRs act as competitive inhibitors of death receptor signaling by death ligands (such as Fas or TRAIL), it is feasible to use the specific antibody of DcRs to enhance the killing effect of immune cells (such as NK cells) on SnCs. In addition, we can take advantage of the CAR-T strategy (Amor et al., 2020) to establish DcR-sepecific CAR T cells to kill SnCs as will be discussed later.

### Surface Molecules and Immunosurveillance for Senolysis

Emerging molecules or proteins specifically present on the surface of SnCs have been discovered, which may play roles in the maintenance and survival of SnCs, targeting of which have been a novel senolytic strategy recently (Rossi and Abdelmohsen, 2021). The surface molecules have been shown to have a major role in the recognition and clearance of SnCs by the immune system. For example, SnCs express ligands for the NKG2D receptor of NK cells including MICA/B and ULBP2, which may drive the recognition and elimination of SnCs by NK-mediated cytotoxicity (Sagiv et al., 2016; Muñoz et al., 2019). SnCs express non-classical MHC molecule HLA-E, which interacts with the inhibitory receptor NKG2A of NK cells and highly differentiated CD8^+^ T cells to inhibit immune responses against SnCs. Accordingly, blocking the interaction between HLA-E and NKG2A boosts immune responses against SnCs (Pereira et al., 2019). Proteomics analysis identified dipeptidyl peptidase 4 (DPP4) as a novel protein marker on the surface of SnCs, targeting of which sensitized SnCs, but not dividing fibroblasts to cytotoxicity by natural killer cells (Kim et al., 2017). Amor et al. identified the urokinase-type plasminogen activator receptor (uPAR) as a cell-surface protein that is upregulated in SnCs *in vitro* and *in vivo* (Amor et al., 2020). They established uPAR-specific chimeric antigen receptor (CAR) T cells (CAR-T) to selectively clear SnCs, which extended the survival of mice with lung adenocarcinoma that were treated with MEK and CDK4/6 inhibitors to induce senescence, ameliorated liver fibrosis and improved liver function by clearing senescent hepatic stellate cells (Amor et al., 2020). In addition, Althubiti et al. have screened a series of the plasma membrane-associated proteins preferentially expressed on the surface of SnCs (Althubiti et al., 2014). The surface molecules and their implications in senolysis have been systematically summarized and reviewed (Rossi and Abdelmohsen, 2021). However, there are still some safety concerns on the use of immune system to eliminate SnCs. Although the surface molecules are highly expressed in SnCs, they can be expressed in proliferating cells, albeit at low levels, leading to side effects (Ravichandra et al., 2021). More researches are needed to verify the senolytic effect and evaluate the safety of immune clearance of SnCs.

## Conclusions and Perspectives

Aging represents a progressive decline in the physiological properties of tissues/organs and the overall fitness of the organism. SnCs accumulate with age and play a casual role in aging as well age-related pathologies. One major characteristic of SnCs is their ability to resist apoptosis induced by different stimuli. For normal cells, increased resistance is an important safety mechanism against acute stress to maintain homeostasis. However, for SnCs, apoptotic resistance can lead to abnormal accumulation, which is detrimental to the organism. Senolytics and other novel strategies (PROTAC, β-galactosidase-modified prodrugs and CAR-T cells) were developed to selectively target SnCs and have shown great potential to delay aging and to treat age-related diseases. It is encouraging to know that several senolytics have been approved for clinical trial and have shown beneficial effects (Niedernhofer and Robbins, 2018; Thoppil and Riabowol, 2019; Kirkland and Tchkonia, 2020). The latest findings that senolytics can be used to prevent or treat virus infection-associated diseases, such as COVID-19 infection, expanding the applications of senolytics in clinic. Most of the current senolytics are discovered by targeting the SCAPs. However, the major challenge in developing senolytics is the high heterogenicity of SnCs, and their diverse origins. SnCs can resist apoptosis via different mechanisms depending on cell type and apoptotic stimuli. In this review, we systematically summarized the proteins or pathways involved in the apoptotic resistance of SnCs, which are not independent but rather interconnected. Inhibitors targeting some of the proteins or pathways have been developed as novel senolytics. However, whether the rest ones are senolytic targets has yet to be investigated. We believe that understanding of the underlying mechanisms for apoptotic resistance of SnCs can help to identify more targets that can be used to develop cell-specific or broad-spectrum therapeutic senolytics to clear SnCs. It should be noted that clearance of SnCs can cause undesirable side effects. For example, continuous or acute elimination of SnCs disrupted blood-tissue barriers with subsequent liver and perivascular tissue fibrosis and health deterioration (Grosse et al., 2020). More caution should be exercised for the systemic use of senolytics for health benefits (Figure 1B). We expect to witness more discoveries in novel and safe senolytics for the use as an intervention to treat SnC-related diseases in the near future.
